# Dl-3-n-butylphthalide attenuates mouse behavioral deficits to chronic social defeat stress by regulating energy metabolism via AKT/CREB signaling pathway

**DOI:** 10.1038/s41398-020-0731-z

**Published:** 2020-02-03

**Authors:** Wei Wang, Ting Wang, Shunjie Bai, Zhi Chen, Xunzhong Qi, Peng Xie

**Affiliations:** 1 NHC Key Laboratory of Diagnosis and Treatment on Brain Functional Diseases, The First Affiliated Hospital of Chongqing Medical University, Chongqing, China; 2 Institute of Neuroscience and the Collaborative Innovation Center for Brain Science, Chongqing Medical University, Chongqing, China; 3 Department of Neurology, The First Affiliated Hospital of Chongqing Medical University, Chongqing, China; 4 Key Laboratory of Laboratory Medical Diagnostics Designated by the Ministry of Education, School of Laboratory Medicine, Chongqing Medical University, Chongqing, China; 5 Department of Laboratory Medicine, The First Affiliated Hospital of Chongqing Medical University, Chongqing, China; 6 Chongqing Key Laboratory of Neurobiology, Chongqing, China; 7 Chongqing Key Laboratory of Cerebrovascular Disease Research, Chongqing, China

**Keywords:** Neuroscience, Diseases

## Abstract

Major depressive disorder (MDD) is a severe mental disorder associated with high rates of morbidity and mortality. Current first-line pharmacotherapies for MDD are based on enhancement of monoaminergic neurotransmission, but these antidepressants are still insufficient and produce significant side-effects. Consequently, the development of novel antidepressants and therapeutic targets is desired. Dl-3-n-butylphthalide (NBP) is a compound with proven efficacy in treating ischemic stroke, yet its therapeutic effects and mechanisms for depression remain unexplored. The aim of this study was to investigate the effect of NBP in a chronic social defeat stress model of depression and its underlying molecular mechanisms. Here, we examined depression-related behavior and performed a targeted metabolomics analysis. Real-time quantitative polymerase chain reaction and western blotting were used to examine key genes and proteins involved in energy metabolism and the AKT/cAMP response element-binding protein (CREB) signaling pathway. Our results reveal NBP attenuates stress-induced social deficits, anxiety-like behavior and despair behavior, and alters metabolite levels of glycolysis and tricarboxylic acid (TCA) cycle components. NBP affected gene expression of key enzymes of the TCA cycle, as well as protein expression of p-AKT and p-CREB. Our findings provide the first evidence showing that NBP can attenuate stress-induced behavioral deficits by modulating energy metabolism by regulating activation of the AKT/CREB signaling pathway.

## Introduction

Major depressive disorder (MDD) is an increasingly common psychiatric disorder that globally affects > 300 million people of all ages, and is also one of the leading causes of disability worldwide^1,2^. Pharmacotherapy is the primary choice for medical management of MDD. Classes of first-line antidepressant medications have been approved to reduce depressive symptoms, but it often takes weeks to produce a measurable benefit. Further, some drugs produce significant side-effects including fatigue, weight gain, nausea, headache, and sexual dysfunction^3–5^. Hence, the search for new treatments of MDD has always been a focus in biomedical research, with investigation of alternative treatments urgently needed to reduce the heavy burden of MDD on patients and society.

Increasing evidence suggests the involvement of energy metabolism pathways in MDD. We have previously shown dysfunction in energy metabolism in most brain regions and plasma in a rodent model of chronic mild stress^6–9^. Corroboratively, our recent study demonstrated the disruption of glucose energy metabolism in naturally occurring depression in cynomolgus monkeys and patients with MDD^10^. At present, energy metabolism abnormalities in depression mainly include: (1) dysfunction of nutrient utilization in glycolysis/gluconeogenesis^11,12^; (2) mitochondrial damage in constituents of the tricarboxylic acid (TCA) cycle^9,13–16^; and (3) functional damage in the mitochondrial respiratory chain^17,18^. Minimizing perturbations in energy metabolism may prevent development of depression, although antidepressants with the relevant properties are not yet clinically available. Thus, targeting energy metabolism pathways may be important for identifying novel antidepressant drugs.

Dl-3-n-butylphthalide (NBP) represents a family of compounds initially isolated from the seeds of *Apium graveolens* Linn (celery), which are approved for the treatment of acute ischemic stroke. Previous studies have shown that NBP improves stroke outcome by protecting mitochondrial function and improving energy metabolism^19–21^. NBP exerts improvements on cerebral energy metabolism by protecting the integrity of mitochondrial structure^22^, increasing activity of mitochondrial complex enzymes^23^, improving activity of mitochondrial ATPase^24^, and maintaining stability of cell membrane potential^25^. Moreover, recent studies have shown that by promoting energy metabolism, NBP may be effective in treating neurological disorders beyond the management of stroke. NBP exhibits protective effects against mitochondrial damage by inhibiting amyloid β (Aβ)-induced mitochondrial dysfunction, whereas Aβ induces active caspase-3, caspase-9, and cytochrome c expression in Alzheimer’s disease^26^. NBP ameliorates SH-SY5Y cell survival against rotenone, and improves mitochondrial membrane potential reductions and reactive oxygen species generation and apoptosis in Parkinson’s disease^27^. MDD is the most frequent psychiatric disorder involving mitochondrial dysfunction and altered energy metabolism^28^, yet no studies investigating whether NBP exerts antidepressant effects have been reported. Accordingly, we administered NBP to mice subjected to chronic social defeat stress (CSDS), a well-validated model of depression, to investigate the antidepressant effects of NBP.

Many studies have focused on signaling pathways involved in regulation of energy metabolism. One of the most studied mediators of these pathways is AKT (also known as protein kinase B)^29–31^. AKT is a serine/threonine kinase and signaling molecule of cell growth and differentiation, which acts as a central node of many signaling pathways. AKT may regulate glucose metabolism by trafficking cellular uptake of glucose and altering gene expression^32^ and the mitochondrial membrane gradient^33–35^. AKT is a phosphoprotein that is capable of phosphorylating a wide range of downstream effectors. Cyclic AMP response element-binding protein (CREB) was shown to be phosphorylated by AKT at Ser133, which increases its binding to CREB-binding protein (CBP) and enhances CREB-mediated transcription of genes that are critical for survival^36^. CREB is a nuclear transcription factor that has an important role in direct transcriptional activation of gluconeogenic genes. Thus, our study investigated whether the effect of NBP administration on energy metabolism was regulated by the AKT/CREB signaling pathway.

Our primary aim was to determine whether NBP administration can modulate or prevent stress-induced behavioral deficits, and in addition, investigate candidate signaling pathways to determine the potential mechanism in the hippocampus (HP) and prefrontal cortex (PFC). Our study may lead to identification of potential therapeutic targets for MDD and be important to antidepressant drug studies.

## Materials and methods

### Animals

Healthy male C57BL/6 J mice (aged 7–8 weeks and weighing 20–23 g, *N* = 40) and male CD1 mice (aged 18–20 weeks and weighing 35–40 g, *N* = 90) were purchased from the Experimental Animal Centre of Chongqing Medical University (Chongqing, China). During the entire study, the mice were maintained in a temperature and humidity controlled room (21–22°C, 55 ± 5%) under a 12 h light/dark cycle with access to food and water freely. The study were approved by the Ethics Committee of Chongqing Medical University.

### Drug treatment

All mice were randomly divided into control with placebo group (CON + PLA group) and CSDS with placebo group (CSDS + PLA group) and CSDS with NBP group (CSDS + NBP group) after 1 week of adaptive feeding. The CON + PLA group (*N* = 12) and CSDS + PLA group (*N* = 15) were treated with soybean oil, and the CSDS + NBP group (*N* = 13) was treated with NBP (60 mg/kg, diluted in soybean oil)^37^. Mice received intragastric administration daily in a volume of 200 μl between 14:00 PM and 14:30 PM for 24 days.

### CSDS

An established CSDS protocol was used to induce depressive-like behavior in mice^38,39^. The CSDS procedure in the study was performed as previously reported^40–42^. The protocol is beginning with the selection of aggressive CD1. Forty-one CD1 mice with appropriate characteristics as described below were selected from 90 CD1. There are two criteria for screening the aggressive behavior of CD1 mice as below: first, CD1 mice must have attacked the C57BL/6 J mice at last 2 consecutive days in the period of 3 days of screening; second, the initial attack on C57BL/6 J must be < 60 seconds during each 180-second screening process per day. House 41 CD1 mice singly in the one side of defeated cages as “resident” to habituate to their new colony facility for 7 days prior to the start of defeat sessions. During the 10-day defeat period, a C57BL/6 J mouse as “intruder” was introduced into the home cage of a novel aggressive “resident” for 10 min every day. After the physical stress, the C57BL/6 J mouse was immediately transferred to the opposite compartment, which was separated by a perforated transparent plexiglass divider for 24 h continued sensory contact. Mice were excluded from the study if repeated defeats lead to open wounds on the C57BL/6 J mice that exceeded 1 cm in length.

### Social interaction test

Social interaction (SI) test was used to detect the social avoidance behavior. Twenty-four hours after the last stress session, the C57BL/6 J mouse was housed individually to perform the test, which was proceeded by placing mice in an open field box (44 cm × 44 cm × 30 cm) with a perforated plastic box (10 cm × 7 cm × 18cm) located at one end and was carried out under the condition of red light. In first detection, the perforated plastic box was empty. The movement of the C57BL/6 J mice was videotaped (Ethovision, Noldus, The Netherlands) for 2.5 min. In second detection, an unfamiliar CD1 was placed in the perforated plastic box and the movement of the C57BL/6 J mice was also recorded for 2.5 min. The SI ratio = time spent in an interaction zone with a CD1 mouse/time spent in an interaction zone without a CD1 mouse. Mice with SI ratio > 1 were defined as “resilient” and mice with SI ratio below 1 were defined as “susceptible”.

### Behavioral testing

After drug treatment, a series of behavioral tests were performed during day time (light-on periods) under conditions of dim light and low noise.

#### Sucrose preference test

The sucrose preference test (SPT) was applied to assess the anhedonia response in rodents. Mice were habituated to 1% sucrose water for 3 days prior to the test. After 24 h deprivation of water and food, mice were provided with 1% sucrose water and pure water. The consumption levels were measured after 24 h testing. Sucrose preference (%) = (sucrose water intake/(sucrose water intake + pure water intake)) × 100.

#### Three-chambered interaction test

The three-chambered interaction test was used to assess social impairment phenotypes in rodents. The test was proceeded in a rectangular, three-chambered box (60 cm × 40 cm × 30 cm), which was divided by two plastic clapboard with a small hole to create optimum entryways and encourage exploration across chamber openings, and two perforated plastic boxes (10 cm × 7 cm × 18 cm) was also required. The testing procedure consists of two phases: In first detection, the three-chambered box was empty, and the movement was not videotaped. In second detection, a perforated plastic box with a stimulus mouse (an unfamiliar C57BL/6 J mouse) and an empty one were placed on the two side of the three-chambered box. The distance traveled, the duration spent and numbers of entrances in the three chambers were recorded for 5 min.

#### Open field test

The open field test (OFT) was used to assess the locomotion activity and exploratory behavior of rodents. Mice were placed in the center of an open field (44 cm × 44 cm × 30 cm), and after 30 s of adaptation, the distance traveled, the duration spent in the center numbers of entrances and rears were recorded during the final 5 min of the test.

#### Elevated plus maze test

The elevated plus maze test (EPM) was used to assess anxiety-like behavior in rodents. The maze was composed of two open arms (30 cm×6 cm) and two closed arms (30 cm × 6 cm × 15 cm). A central 6 cm × 6 cm square platform had access to all arms. Mice were placed in the center of the maze, habituated for 30 s and recorded the distance traveled, the number of entries and the duration in each arm in the next 5 min.

#### Light/dark transition test

The light/dark transition test was a method of evaluating anxiety-like behavior in rodents. Mice were placed in the light zone of an open field (20 cm×15 cm × 25 cm), which were same as the dark zone, except that the latter has a cover to keep away from light. The two zones were divided by a black plastic clapboard with a small hole to create optimum entryways. The distance and time traveled in light zone, numbers of transition were recorded during the 5 min testing.

#### Y-maze

The Y-maze was applied to evaluate spatial recognition memory in rodents. The Y-maze apparatus was composed of three arms (30 cm × 6 cm× 15 cm) intersecting at 120°. Mice were placed in the center of the maze and allowed to freely explore the three arms for 8 min. The sequence and total numbers of entering into three arms were noted. The percentage of spontaneous alternation (%) = ((number of alternations)/(total number of arm entries − 2)) × 100.

#### Tail suspension test

The TST was used to evaluate behavioral despair and reflected by the duration of immobility. Mice were individually suspended by their tails using a small piece of adhesive tape, which was placed 2 cm from the tip of the tail. The test lasted for 6 min. The duration of immobility was analyzed during the final 5 min. Animals were considered to be immobile only when they remained suspended passively and were completely motionless. A trained observer blinded to group recorded the total immobility time. This test was real-time monitored with a video surveillance system.

### Sample preparation

After the behavioral tests, mice were killed by decapitation after anesthesia. The entire brain of each mouse was removed, and the HP and PFC tissues were separated. All tissues were quickly frozen in liquid nitrogen and then stored at −80 °C until analysis. After being accurately weighed, the brain sample was transferred into precooling EP tube and homogenized with 200 μl precooling ultrapure water, and then added into 800 μl precooling methanol/acetonitrile solution (1:1, v/v) and vortex-mixed. Next, after 20 min ice-water bath ultrasonic extraction, the mixture was incubated at −20 °C for 1 h to precipitate protein, then was centrifuged at 14,000 × *g*, 4 °C for 20 min. The supernatant was transferred into a new EP tube and vacuum dried. Before the detection, 100 μl acetonitrile/H_2_O solution (1:1, v/v) was added to the dried extract to dissolve, and centrifuged at 14,000 × *g*, 4 °C for 20 min. Then the supernatant was used for the LC–MS/MS analysis.

### LC–MS/MS analysis

LC–MS/MS was conducted with an Agilent 1290 Infinity chromatography system and AB SCIEX QTRAP 5500 mass spectrometer. In all, 10 mm ammonium acetate solution and acetonitrile were used as mobile phase A and B. The samples were placed in 4 °C automatic sampler with column temperature of 45 °C, and the flow rate was 0.3 mL/min and the injection volume was 2 μl. The gradient of mobile phase B was as follows: 0–18 min at 90–40% acetonitrile; 18–18.1 min at 40–90% acetonitrile and 18.1–23 min at 90%. Besides, the MS system was operated with a negative ion mode under the following conditions: ion sapary voltage floating, −4500 V; source temperature, 450 °C; ion source gas 1, 45; ion source gas 2, 45; curtain gas, 30. Analyses were determined by electrospray ionization using multiple reaction monitoring. Peak chromatographic area and retention time were analyzed with Multiquant software. The standard substance of energy metabolites was used to calculate the retention time and identify metabolites.

### RNA extractions, reverse transcription, and real-time quantitative PCR

The HP and PFC samples stored at −80 °C were taken out to unfreeze. In all, 1 μg of RNA, isolated from the each tissue in TRIzol (Invitrogen, USA), was used for cDNA synthesis by PrimeScript RT reagent Kit (Takara, Japan) following the manufacturer’s protocol. SYBR gene detection system (Roche, Germany) was used to determine specific gene expression levels in each tissue. Each transcript value was calculated as the average of triplicate samples across experimental condition. Values were normalized to β-actin and GAPDH. Gene expression was calculated using the formula 2^−△△t^. The primers are shown in Supplementary Table S1.

### Western blotting

The HP and PFC tissues were dissociated by radioimmunoprecipitation assay solution containing phosphatase inhibitor and protease inhibitor cocktail (Roche, Germany) and processed by ultrasound, finally centrifuged at 14,000 × *g*, 4 °C for 15 min and collected the supernatant to obtain the protein solution. The proteins in the sample were separated using a 10–12% SDS polyacrylamide gel, then transferred to polyvinylidene fluoride membrane (Millipore, USA) for blocking. The proteins were incubated with rabbit monoclonal anti-AKT1/2/3 (Abcam, ab179463,1:5000), rabbit monoclonal anti-phospho-AKT (Cell Signaling, #4060, 1:1000), rabbit monoclonal anti-CREB (Cell Signaling, #9197,1:1000), rabbit monoclonal anti-phospho-CREB (Ser133) (Cell Signaling, #8212, 1:1000), rabbit monoclonal anti-tropomyosin receptor kinase B (Trkb) (Cell Signaling, #4603,1:1000), rabbit monoclonal anti-brain-derived neurotrophic factor (BDNF) (Abcam, ab108319,1:1000), rabbit monoclonal anti-SDHc (Abcam, ab155999, 1:1000), rabbit monoclonal anti-Sucla2 (Abcam, ab183513, 1:5000), rabbit polyclonal anti-P2rx1 (Bioss, bs-12107R, 1:1000), rabbit monoclonal anti-GAPDH (Abcam, 1:8000) and mouse monoclonal anti-β-tublin (Cell Signaling, ab181602,1:8000) overnight at 4 °C and subsequently incubated with secondary anti-rabbit antibodies (Bio-rad, cat# 170–6515,1:10,000) or secondary anti-mouse antibodies (Bio-rad, cat# 170–6516,1:10,000) for 2 h at room temperature. The signals were visualized using a chemiluminescence kit (Millipore, USA) and analyzed with Quantity one software (Bio-Rad, USA).

### Statistics

All data were analyzed using SPSS 21.0 (IBM, USA) and values were considered significant at *p* < 0.05. The statistic differences among three groups were assessed by one-way analysis of variance (ANOVA) followed by Fisher’s least significant difference correction. Data were expressed as mean ± SEM or mean. The results of statistical analyses were shown by Graphpad Prism 7.0 (San Diego, USA). Pearson’s correlation coefficient was applied to calculate the correlations between behaviors and metabolites in the HP and PFC.

## Results

### NBP administration prevents development of social avoidance in CSDS

**Fig. 1:**
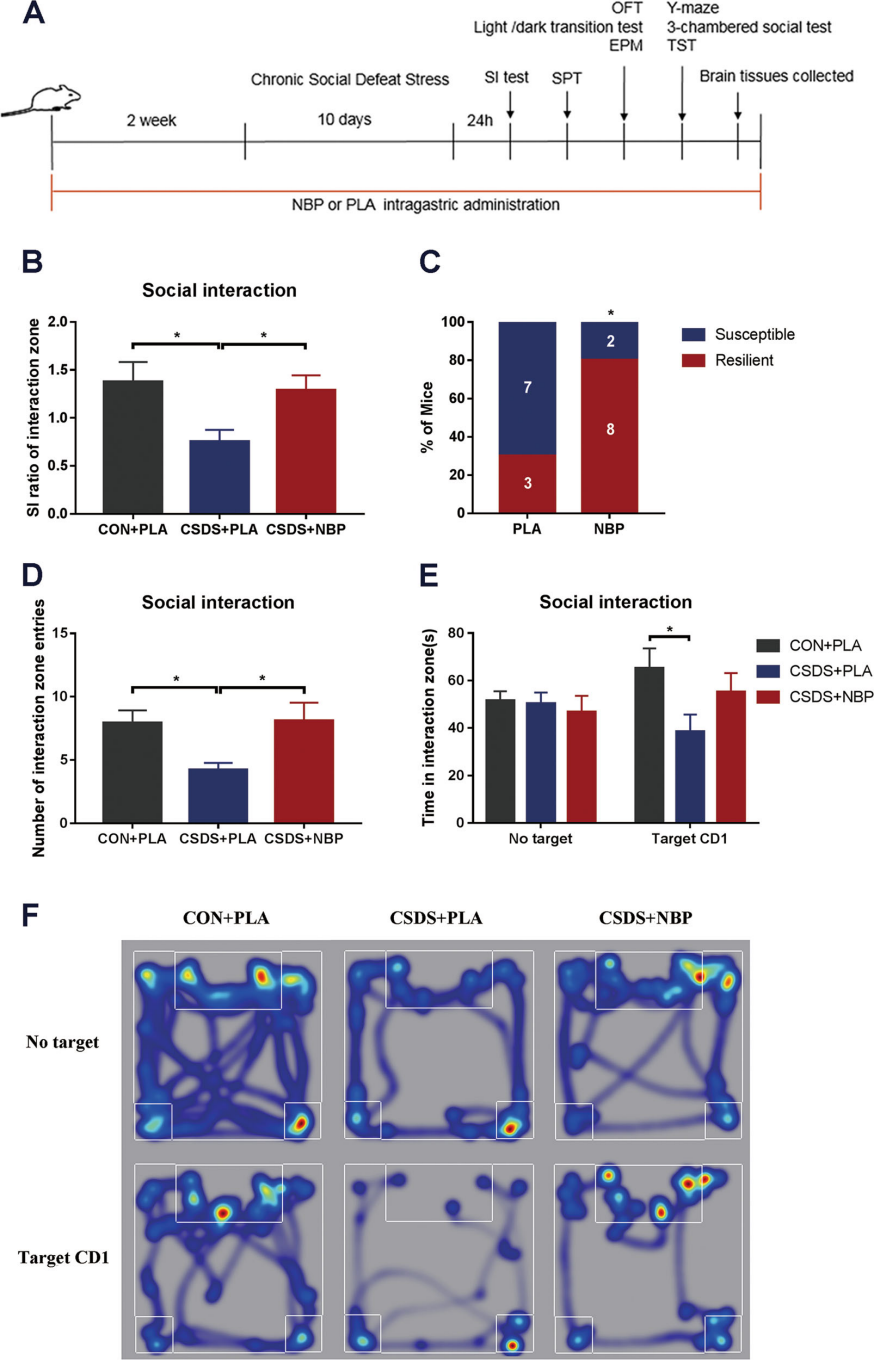
Results of the social interaction test after drug administration (NBP and PLA) and chronic social defeat stress. **a** Schedule of the experimental procedures. **b** SI ratios of interaction zones in CON + PLA, CSDS + PLA, and CSDS + NBP mice. **c** Percentage of mice categorized as resilient or susceptible following PLA (*N* = 10) or NBP (*N* = 10) administration. The number of animals is indicated inside the bar graph. Mice with SI ratio > 1 were categorized as resilient. **d** Number of entries into interaction zone. **e** Time spent in interaction zone. **f** Heat maps showing representative tracks of mice from each group in the social interaction test (CON + PLA group: *N* = 12; CSDS + PLA group: *N* = 11; CSDS + NBP group: *N* = 11). *p < 0.05. Data represent mean ± SEM. *NBP* dl-3-n-butylphthalide, *PLA* placebo, *SI* social interaction test, *SPT* sucrose preference test, *OFT* open field test, *EPM* elevated plus maze, *TST* tail suspension test.

As shown in the experimental approach schedule (Fig. 1a), we used the social interaction test to determine the efficacy of the CSDS model and determine whether NBP promotes resilience to CSDS in mice. We found lower SI ratios in the CSDS + PLA group than the CON + PLA group, but higher SI ratios in the CSDS + NBP group compared with the CSDS + PLA group (Fig. 1b). Of the mice showing defeat, a significantly higher proportion of NBP mice (80%) exhibited resilience to stress compared with PLA mice (30%) (*χ*^2^ [1] = 5.051, *p* = 0.025) (Fig. 1c). Moreover, ANOVA detected a significant difference in interaction zone entries: post hoc comparisons showed that CSDS + PLA mice entered interaction zones less times than CON + PLA mice, whereas CSDS + NBP mice entered center zones more times than CSDS + PLA mice (Fig. 1d). Interestingly, as shown by time spent in interaction zones and typical tracks (Fig. 1f), CSDS + PLA mice spent less time in interaction zones compared with CON + PLA mice when a target CD1 was present. Conversely, there were no difference in time spent in interaction zones among the three groups when a target CD1 was absent (Fig. 1e).

### NBP treatment ameliorates decreased body weight and increases sociability of CSDS

**Fig. 2:**
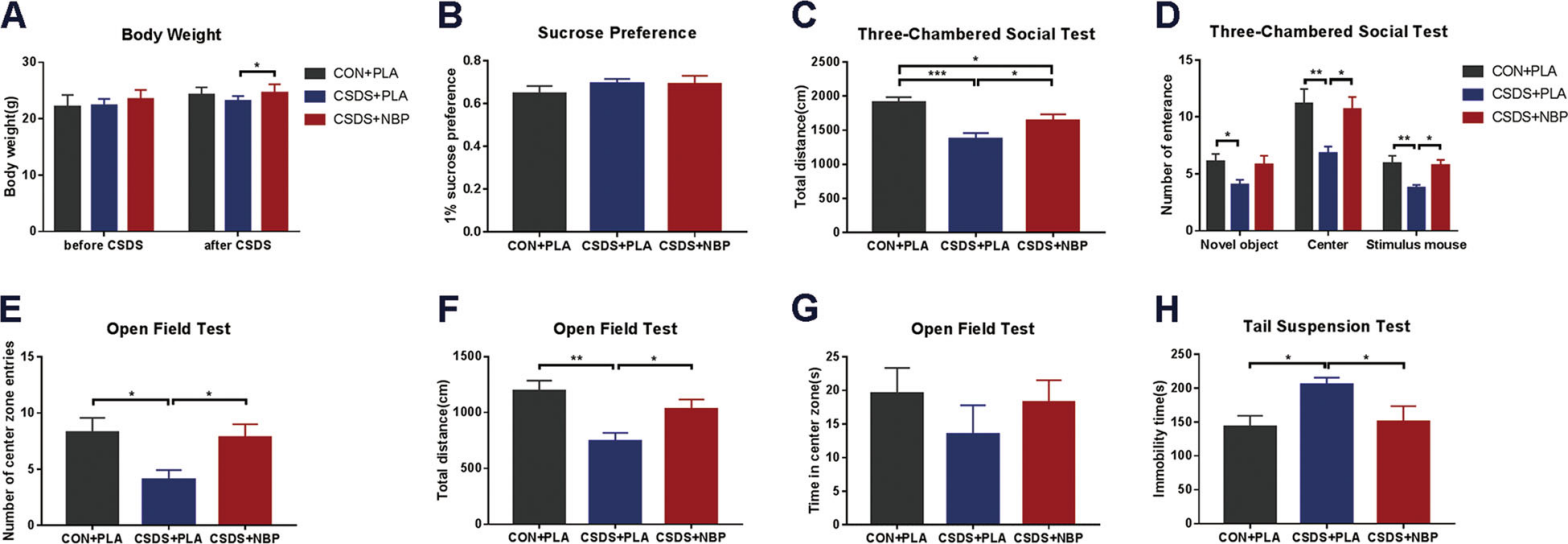
Effect of drug treatment and chronic social defeat stress on body weight, three-chambered social test, anxiety-like behavior, and despair behavior. **a** Body weight before and after chronic social defeat stress among the three groups. **b** 1% sucrose preference among the three groups. **c** Total distance traveled in the three chambers in three-chambered interaction test. **d** Number of entries into each of three chambers in three-chambered interaction test. **e** Number of center zone entries after chronic social defeat stress (CSDS) in the open field test (OFT). **f** Total distance after CSDS in the OFT. **g** Time in the center zone in the OFT. **h** Immobility time in the tail suspension test (CON + PLA group: *N* = 12; CSDS + PLA group: *N* = 11; CSDS + NBP group: *N* = 11). *p < 0.05, **p < 0.01, and *** *p* < 0.001. Data represent mean ± SEM.

Before the defeat procedure, mice in all three groups showed no statistical difference in body weight, as expected. However, after 10 days of the defeat procedure, body weight was different among the three groups (Fig. 2a). Post hoc comparisons showed that mice in the CSDS + NBP group weighed more (24.56 ± 1.47 g) compared with the CSDS + PLA group (23.15 ± 0.81 *g*), whereas mice in the CON + PLA group (24.18 ± 1.32 g) showed no difference compared with the other two groups. The SPT showed no significant difference in sucrose preference among the three groups (Fig. 2b). However, sucrose water intake was less in CSDS + NBP mice than CON + PLA mice, although there was no difference in pure water intake (see Supplementary Fig. S1).

In the three-chambered interaction test, there was a significant difference in total distance among the three groups (Fig. 2c). Mice in the CSDS + PLA group showed significantly less movement in the three chambers than mice in the CON + PLA group, whereas total distance in the CSDS + NBP group increased markedly compared with the CSDS + PLA group. Further, the number of mice entering the stimulus mouse chamber showed significant differences among the three groups. Post hoc testing showed a reduced number of mice entering the stimulus mouse chamber in the CSDS + PLA group compared with the CON + PLA group. Meanwhile, the number increased in the CSDS + NBP group compared with the CSDS + PLA group (Fig. 2d).

### NBP attenuates anxiety-like and despair behavior following CSDS

In the OFT, CSDS + PLA group mice showed less locomotion activity and anxiety-like behavior compared with CON + PLA group mice, reflected by a marked decrease in number of center zone entries and total distance (Fig. 2e, f). Interestingly, the CSDS + NBP group showed recovery of exploration and locomotion, reflected by an increase in number of center zone entries and total distance compared with the CSDS + PLA group (Fig. 2e, f). Nonetheless, NBP treatment did not affect time spent in the center zone or distance in the center zone and periphery zone or number of rears (Fig. 2g; Supplementary Fig. S2A–C). The EPM and light/dark transition test (see Supplementary Fig. S2D, E) are also widely used to examine anxiety. However, we found no significant differences in these tests. Simultaneously, we performed the TST to detect behavioral despair (Fig. 2h). Immobility time was significantly increased in the CSDS + PLA group compared with the CON + PLA group in the TST, whereas NBP-treated (CSDS + NBP) depressed mice showed less immobility time than PLA-treated (CSDS + PLA) depressed mice. In addition, we found no statistically significant differences in spatial working memory in the Y-maze test (see Supplementary Fig. S2F).

### NBP treatment alters metabolite levels of energy metabolism pathways in the HP and PFC of mice

There were significant differences in the levels of target metabolites among the three groups in the HP and PFC. These differential metabolites are shown in Supplementary Table S2.

**Fig. 3:**
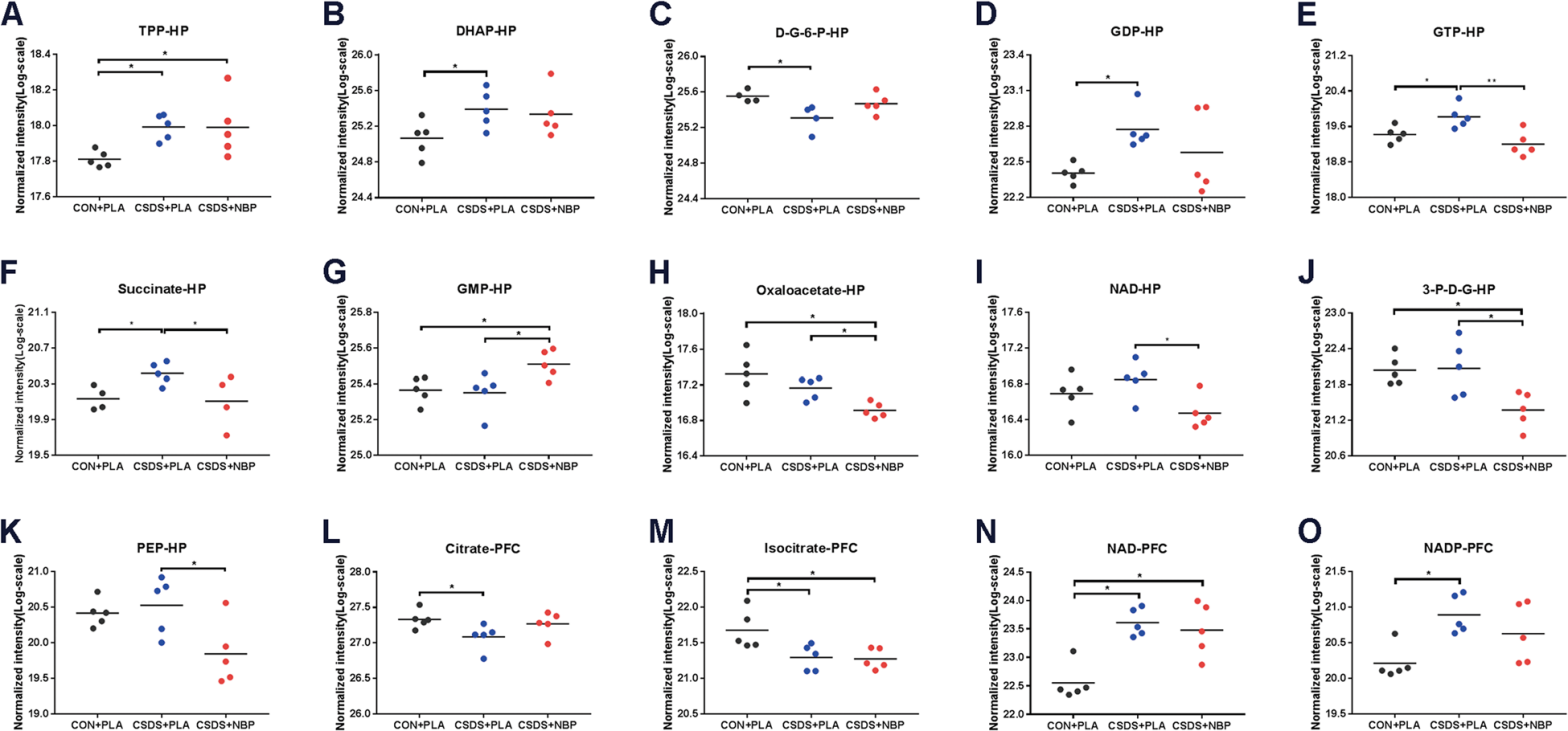
Differential target metabolites in the hippocampus and prefrontal cortex of CSDS model mice. **a**–**k** Levels of thiamine pyrophosphate (TPP) **a**, dihydroxyacetone phosphate (DHAP) **b**, d-glucose-6-phosphate (d-G-6-P) **c**, guanosine 5’-diphosphate (GDP) **d**, guanosine 5’-triphosphate (GTP) **e**, succinate **f**, guanosine monophosphate (GMP) **g**, oxaloacetate **h**, nicotinamide adenine dinucleotide (NAD) **i**, 3-phospho-d- glycerate (3-P-d-G) **j**, and phosphoenolpyruvate (PEP) **k** were calculated by LC–MS/MS in hippocampal (HP) extracts from CON + PLA, CSDS + PLA, and CSDS + NBP mice. **l**–**o** Levels of citrate **l**, isocitrate **m**, NAD **n**, and nicotinamide adenine dinucleotide phosphate (NADP) **o** were also detected in the prefrontal cortex (PFC) (*N* = 5 mice per group). *p < 0.05 and **p *<* 0.01.

In the HP, compared with the CON + PLA group, thiamine pyrophosphate (TPP; Fig. 3a), dihydroxyacetone phosphate (DHAP; Fig. 3b), guanosine 5’-diphosphate (GDP; Fig. 3d), guanosine 5’-triphosphate (GTP; Fig. 3e), and succinate (Fig. 3f) were all significantly increased, whereas d-glucose-6-phosphate (d-G-6-P; Fig. 3c) was decreased in the CSDS + PLA group. In addition, guanosine monophosphate (GMP; Fig. 3g) showed a significant increase in the CSDS + NBP group compared with the CSDS + PLA group. Meanwhile GTP (Fig. 3e), succinate (Fig. 3f), oxaloacetate (Fig. 3h), nicotinamide adenine dinucleotide (NAD; Figs. 3i), 3-phospho-d-glycerate (3-P-d-G; Fig. 3j), and phosphoenolpyruvate (PEP; Fig. 3k) levels were significantly reduced in the CSDS + NBP group. There were no significant differences in other metabolites in the HP. In the PFC, citrate (Fig. 3l) and isocitrate (Fig. 3m) were reduced in the CSDS + PLA group compared with the CON + PLA group, while NAD (Fig. 3n) and nicotinamide adenine dinucleotide phosphate (NADP; Fig. 3o) were both significantly increased in the CSDS + PLA group. There were no significant differences in other metabolites in the PFC.

A total of 11 different metabolites were significantly altered in the HP. Compared with the CON + PLA group, five metabolites (TPP, DHAP, GDP, GTP, succinate) were increased and one metabolite (d-G-6-P) decreased in the CSDS + PLA group. Meanwhile six metabolites (GTP, succinate, NAD, PEP, oxaloacetate, 3-P-d-G) were decreased and one metabolite (GMP) increased in the CSDS + NBP group compared with the CSDS + PLA group. Further, a total of four different metabolites were significantly altered in the PFC. Compared with the CON + PLA group, two metabolites (citrate, isocitrate) were decreased and two metabolites (NAD, NADP) increased in the CSDS + PLA group. Among the differentially altered metabolites, only NAD was altered in both the HP and PFC, with the others metabolites altered in only one brain region (HP or PFC). This indicates that the altered metabolites show significant brain region specificities.

### Differential metabolites in the HP and PFC correlate with behavior

Correlation analysis revealed that differential metabolites in the HP and PFC correlate with observed behavior (see Supplementary Fig. S3, S4). In the HP, a significantly negative association was shown between succinate levels and interaction zone SI ratio (*r* = −0.565, *p* *=* 0.028; Fig. S4a) and interaction zone entries (*r* = −0.569, *p* = 0.027; Fig. S4b). Negative association was also shown between GMP and novel object zone distance in the three-chambered test (*r* = −0.561, *p* = 0.029; Fig. S4c). Significant positive association was shown between 3-P-d-G levels and novel object zone distance in the three-chambered test (*r* = 0.520, *p* = 0.047; Fig. S4d). Similarly, positive association was shown between PEP levels and novel object zone distance in the three-chambered test (*r* = 0.600, *p* *=* 0.018; Fig. S4e). For oxaloacetate, there was positive association with stimulus mouse zone distance (*r* = 0.578, *p* *=* 0.024; Fig. S4f) and total distance (*r* = 0.527, *p* = 0.043; Fig. S4g) in the three-chambered interaction test. In the PFC, citrate showed a significantly negative association with novel object zone distance in the three-chambered test (*r* = −0.525, *p* = 0.044; Fig. S4h), but a positive association with interaction zone SI ratio (*r* = 0.526, *P* = 0.044; Fig. S4i). Reduced isocitrate levels after CSDS showed significant positive association with reduced center zone distance in the OFT (*r* = 0.668, *p* = 0.006; Fig. S4j).

### NBP treatment alters gene expression of TCA enzymes and purinergic receptors in the HP and PFC

**Fig. 4:**
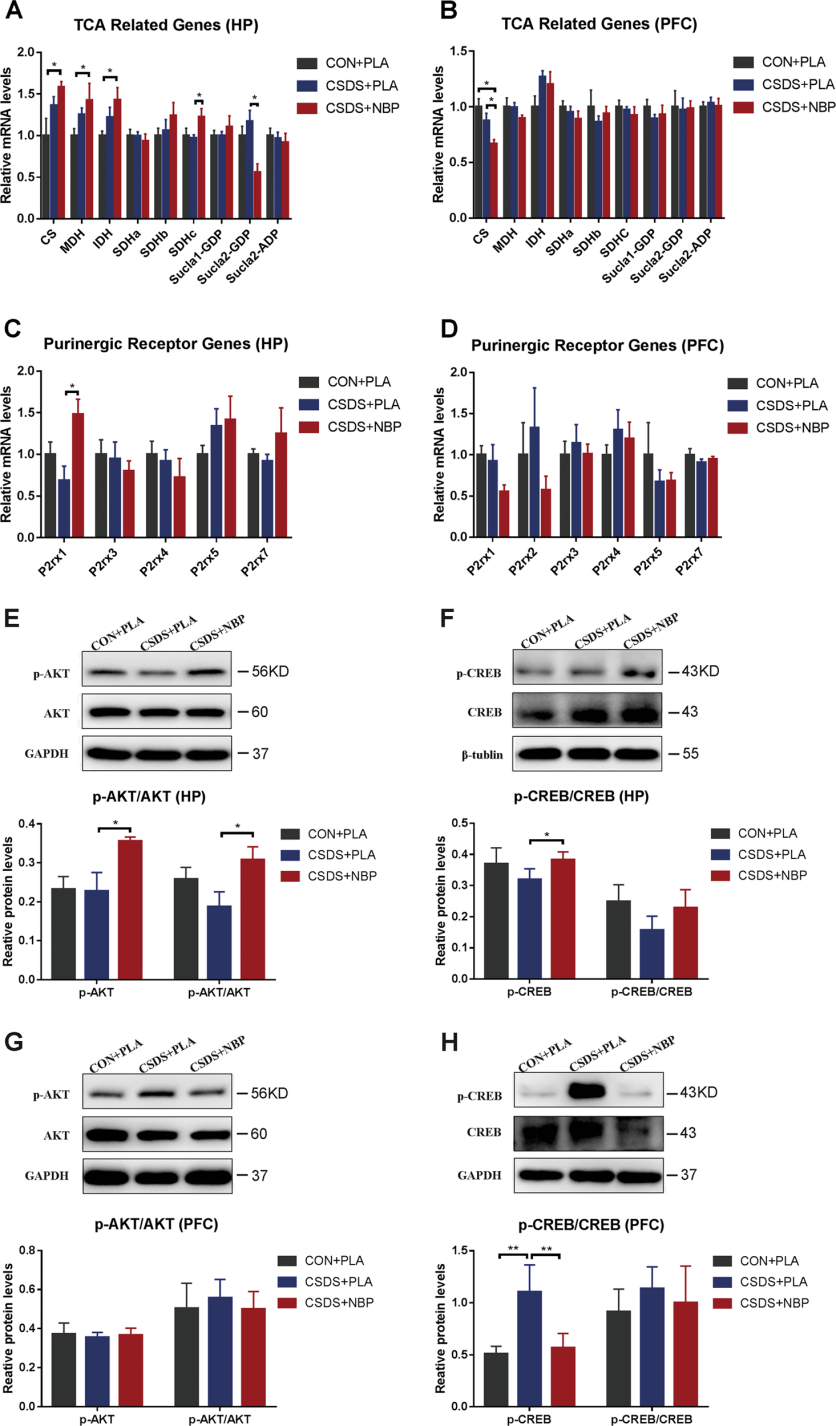
NBP treatment alters mRNA expression levels of key TCA cycle enzymes and purinergic receptors and AKT and CREB protein expression levels in the hippocampus and prefrontal cortex. **a**, **b** mRNA expression levels of key TCA cycle enzymes genes in the hippocampus (HP) **a** and prefrontal cortex (PFC) **b**. **c**, **d** mRNA expression levels of purinergic receptor genes in the HP **c** and PFC **d**. **e** Protein expression levels of p-AKT and p-AKT/AKT in the hippocampus (HP). **f** Protein expression levels of p-CREB and p-CREB/CREB in the HP. **g** Protein expression levels of p-AKT and p-AKT/AKT in the prefrontal cortex (PFC). **h** Protein expression levels of p-CREB and p-CREB/CREB in the PFC (*N* = 6 mice per group). *p *<* 0.05 and **p *<* 0.01. Data represent mean ± SEM. Abbreviations: *CS* citrate synthase, *MDH*, malate dehydrogenase; IDH, isocitrate dehydrogenase; SDHa, SDHb, and SDHc, succinate dehydrogenase complex, subunit A, B, and C; Sucla1-GDP, succinate-Coenzyme A ligase, GDP-forming, alpha subunit; Sucla2-GDP, succinate-Coenzyme A ligase, GDP-forming, beta subunit; Sucla2-ADP, succinate-Coenzyme A ligase, ADP-forming, beta subunit, *P2rx1, P2rx2, P2rx3, P2rx4, P2rx5, and P2rx7* purinergic receptor P2X, ligand-gated ion channel 1, 2, 3, 4, 5, and 7.

Gene expression levels of key TCA cycle enzymes and purinergic receptors were tested by RT-qPCR. In the HP, mRNA levels of SDHc (Fig. 4a) and ligand-gated ion channel 1 of purinergic receptor P2X (P2rx1; Fig. 4c) were significantly increased in the CSDS + NBP group compared with the CSDS + PLA group. Meanwhile, mRNA levels of the beta subunit of succinate-coenzyme A ligase, GDP-forming (Sucla2-GDP) were reduced in the CSDS + NBP group compared with the CSDS + PLA group (Fig. 4a). In the PFC, transcription of citrate synthase (CS) was significantly reduced in the CSDS + NBP group compared with the CSDS + PLA group (Fig. 4b). Alternatively, there were no significant differences in purinergic receptor genes in the PFC (Fig. 4d). We also examined expression of stress-related and serotonin-related genes, but found no statistically significant change in either the HP or PFC (see Supplementary Fig. S5).

### NBP administration increases protein expression of AKT/CREB in the HP

Protein expression levels of AKT, phospho-AKT, CREB, and phospho-CREB were detected by western blotting. In the HP, we found significantly increased expression of p-AKT, p-AKT/AKT (Fig. 4e), and p-CREB (Fig. 4f) in the CSDS + NBP group compared with the CSDS + PLA group. In the PFC, p-CREB levels were increased in the CSDS + PLA group compared with the CON + PLA group, whereas the CSDS + NBP group showed a marked reduction in p-CREB levels (Fig. 4h) compared with the CSDS + PLA group. Protein expression of SDHc, Sucla2, P2rx1, BDNF, and Trkb were also detected in the HP and PFC, but no significant differences were found among the three groups (see Supplementary Fig. S6, S7).

## Discussion

MDD is a common debilitating mental disorder and its etiology is complex and still unknown. Alterations of the HPA axis, hypofunction of monoamine neurotransmitters and glutamatergic system^43^, changes in synaptic plasticity^44^, and disturbances in amino-acid metabolism^45^ and lipid metabolism^46^ have been implicated in the pathogenesis of depression. Comparing the metabolic profiles of patients with MDD, CSDS models and antidepressant of our previous study (see Supplementary Fig. S8) suggested perturbations of energy metabolism have been linked to depression, however, whether modulating energy metabolism may prevent development of the disorder and the possible underlying molecular mechanisms are not known. In our study, we found that NBP treatment ameliorated stress-induced behavioral deficits, which may affect energy metabolism in the brain by regulating the AKT/CREB signaling pathway.

CSDS model is the type of stress consisting of a mixture of physical and psychological components and it shows predictive validity to responds chronic administration of antidepressants^38,39,47–49^. According to the standardized protocol for CSDS in mice, the SI test is used to evaluate whether the model was built successfully, and reduction of SI ratio in defeated mice could reflect the success of the CSDS model in the present study. Surprisingly, we found that NBP administration prevented development of social avoidance, which significantly increased SI ratio in the SI test. Similar results were found in the three-chambered interaction test, which was used to examine sociability in mice. Total distance and number of entries into the stimulus mouse zone were markedly increased after NBP treatment, showing that mice treated with NBP exhibit an increase of exploration and sociability. The SPT is commonly used in assessment the anhedonia of MDD model, whereas the sucrose preference showed no significant difference in our study. Similarly, several previous experiments in our and other groups also found no difference in sucrose preference after the CSDS procedure^40,47,50,51^. Sucrose preference has also been shown to be prone to bias, because taste and smell disturbances, context of liquid intake, and group comparisons can all modify the preference index^50^. In this study, it may be the intragastric administration that affect the taste and smell and cause the sucrose preference unaffected in a stressful situation. The OFT is applied to analyze locomotion and anxiety-like behavior in rodents^52,53^. NBP administration significantly increased total distance traveled, indicating amelioration of locomotion deficits. In addition, in the OFT, there was an increase in number of center zone entries in the NBP-treated mice, suggesting NBP promoted greater exploratory behavior and ameliorated anxiety-like behavior. However, NBP did not influence other anxiety-related behaviors, such as the EPM and light/dark transition test. Therefore, NBP may have a partial anxiolytic effect. After NBP treatment, immobility time decreased in the TST, indicating that NBP impacts despair behavior in mice.

**Fig. 5:**
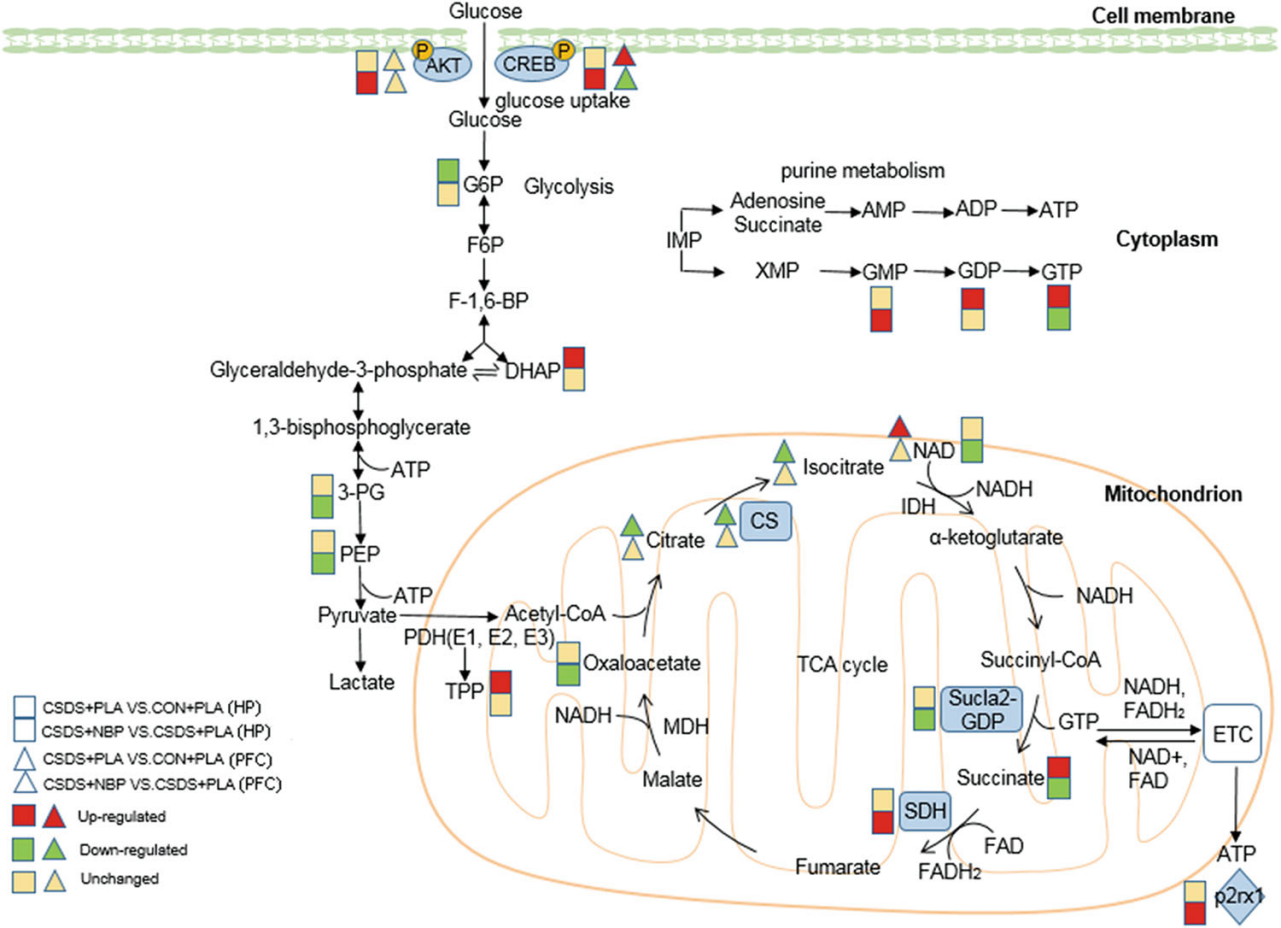
Overview of identified metabolic changes and altered enzymes and purinergic receptor levels, and active targets of p-AKT and p-CREB involved in energy metabolism in the hippocampus and prefrontal cortex. Rectangles indicate metabolic changes in the hippocampus (HP), whereas triangles indicate metabolic changes in the prefrontal cortex (PFC). Upper rectangles and triangles indicate changes in the CSDS + PLA group compared with the CON + PLA group. Lower rectangles and triangles indicate changes in the CSDS + NBP group compared with the CSDS + PLA group. Red represents upregulation, green represents downregulation, and yellow represents no significant change. Abbreviations: *TCA* tricarboxylic acid, *G6P* glucose-6-phosphate, *F6P* fructose-6-phosphate, *F-1 6-BP* fructose-6- biphosphate, *DHAP* dihydroxyacetone phosphate, *3-PG* 3-phospho-glycerate, *PEP* phosphoenolpyruvate, *ATP* adenosine triphosphate, *TPP* thiamine pyrophosphate, *NAD* nicotinamide adenine dinucleotide, *NADH* reduced form of nicotinamide adenine dinucleotide, *GTP* guanosine triphosphate, *FADH2* reduced flavin adenine dinucleotide, *CS* citrate synthase, *IDH* isocitrate dehydrogenase, *Sucla2-GDP* beta subunit of succinate-coenzyme A ligase GDP-forming, *MDH* malate dehydrogenase, *IMP* inosine monophosphate, *AMP* adenosine 5’-monophosphate, *ADP* adenosine diphosphate, *GMP* guanosine monophosphate, *GDP* guanosine diphosphate, *CREB* cAMP response element-binding protein, *P2rx1* purinergic receptor P2X, ligand-gated ion channel 1.

Metabolomics is a widely used tool for exploring molecular pathways underlying symptoms of depression and recovery after pharmacotherapy^54–57^. Levels of energy metabolism-associated metabolites were significantly perturbed in the social defeat mice. A similar disturbance of energy metabolism was also found in our previous studies in chronic unpredictable mild stress, learned helplessness, and chronic restraint stress model^58^. The altered metabolites of energy metabolism pathway are involved in glycolysis and the TCA cycle. Glycolysis, beginning with the phosphorylation of glucose as the primary carbon source, is coupled to the TCA cycle to fully oxidize glucose and supply energy for the brain. Decreased d-G-6-P levels observed in the HP of defeated mice may result from a reduction of brain glucose uptake, which has also been shown in our previous studies of MDD^11,12,59^. Lower levels of d-G-6-P indicate decreased upstream glucose and reduced glycolysis, which is consistent with the changed metabolism of Borna disease virus laboratory Strain V (BDV Strain V) infected rat cortical neurons from our group’s previous study. However, in the virus study, d-G-6-P and other upstream sugars were increased in a natural human strain BDV Hu-H1 cells. These inconsistent results reflected divergent effects of the two virus strains upon glucose metabolism in rat cortical neurons^60^. DHAP and glyceraldehyde 3-phosphate are isomeric, and can transform one another using triose phosphate isomerase. Increased content of DHAP in defeated mice indicates decreased consumption of glyceraldehyde 3-phosphate, suggesting that the glycolysis process is inhibited in defeated subjects. TPP is a coenzyme of the pyruvate dehydrogenase complex, which catalyzes pyruvate oxidation decarboxylation to form acetyl CoA, which enters the TCA cycle. In our research, TPP levels were increased in stress-treated mice, which may provide an explanation for greater carbon flux through the TCA cycle^14^. Levels of TCA metabolite (e.g., succinate, GDP, and GTP) showed significant upregulation in defeated mice in the HP. Succinate is the metabolic component after succinyl CoA synthetase (Sucla2) activity, and a precursor to succinate dehydrogenase (SDH) (Fig. 5). Sucla2 catalyzes the conversion of succinyl CoA into succinate, which is accompanied by generation of GTP via substrate level phosphorylation. This finding is consistent with increased levels of urinary succinate in MDD from our previous study^14^, which may indicate that during depression, individuals consume greater carbon through the TCA cycle. And lower levels of glycolytic intermediates and upstream sugars in conjunction with higher of TCA intermediates indicate an equilibrium shift away from glycolysis and increased carbon flux through the TCA cycle, like the finding in the aforementioned research of BDV Strain V. While, in Hu-H1-infected neurons, lower levels of glycolytic and TCA intermediates combined with higher upstream sugar levels indicated a shift towards gluconeogenesis^60^. These previous results may imply that virus strains might affect different metabolic pathways and elucidating the differences in specific mechanisms requires further investigation. Furthermore, we found significantly decreased citrate and isocitrate levels in the PFC of the defeated group. Citrate and isocitrate are upstream metabolites of the TCA cycle. Reductions may indicate that the process is downregulated in the PFC of defeated mice. Initial studies by our group found that citrate was differentially expressed in male patients with MDD compared with corresponding healthy controls. This was not found in female patients with MDD^61^. This suggests that citrate could be used as a sex-specific urinary metabolite biomarker for diagnosing MDD.

We observed an increase in the glycolysis process with chronic treatment of NBP. Generation of 3-P-d-G is a reversible reaction catalyzed by phosphoglycerate kinase. Alteration of downstream metabolites suggests that NBP treatment might affect glycolysis. Conversion of PEP to pyruvate is catalyzed by pyruvate kinase along with generation of ATP, which is the last step in glycolysis. Downregulation of PEP suggests that the step is enhanced to generate more pyruvate and energy production after NBP treatment. Based on a candidate drug analysis, a recent review suggested that pyruvate may be a candidate drug for the treatment of MDD^62^. Together with changes in diverse glycolytic metabolites, this also suggests that improvement of glycolysis might be the molecular mechanism responsible for the antidepressant-like effect of NBP. Simultaneously, we found decreased TCA cycle intermediate levels after NBP treatment. Significantly decreased levels of succinate and GTP and increased precursor (i.e., GMP) are consistent with decreased Sucla2-GDP and increased SDHc gene expression levels in the NBP group. NAD + is considered to be a coenzyme of many dehydrogenases with crucial roles in energy metabolism and electron transfer. Reduction of NAD + may indicate that enzyme activity and electron transfer are decreased. In addition, oxaloacetate levels (which competitively inhibit SDH in the brain) were significantly decreased after NBP treatment^63^. Reduction of oxaloacetate levels might compensate for a decrease in SDH activity. Thus, we can conclude that chronic pre-treatment with NBP might decrease metabolite levels to generate less carbon through the TCA cycle and achieve a natural recovery. Regulation of aerobic glycolysis and the TCA cycle is the consequence of energy adaptations. With sufficient energy, aerobic oxidation is reduced to save resources. These same results were found with paroxetine treatment^64^ and ketamine treatment^65^.

Besides enzymes in the TCA cycle, we also detected expression of P2X receptors, cation channels gated by ATP, to determine whether purinergic receptors are involved in metabolite responses. Interestingly, mRNA levels of Sucla2-GDP, SDHc, and P2rx1 were significantly altered in the HP, but protein levels showed no significant variation. This may be owing to the regulation of transcriptional or post-translational protein modification, and it may require the increase sample size and other protein-based techniques to validate in further study. Moreover, this finding might also indicate the increased glucose uptake and decreased TCA cycle metabolite levels are not merely regulated by altered TCA cycle enzymes and purine receptors levels.

Recent studies have reported that NBP exerts antioxidant, anti-inflammation, and anti-apoptosis through regulation PI3K/AKT/Nrf2 activation^66,67^ and possible inhibition TLR4/NF-kB signaling pathway^68^. However, the signaling pathways involved in NBP administration in regulating the metabolism have not yet been elucidated. AKT/CREB is a classical signaling pathway involved in numerous biological processes. AKT plays a central role in the regulation of energy metabolism via mechanisms involving, but not fully restricted to, the cellular uptake and utilization of glucose. The phosphorylation and activation of AKT leads to a cascade of insulin signaling events that coordinate trafficking of glucose transporters (GLUT)−4 to the plasma membrane^69^. The similar results have been observed in the studies of human cancers. The increased capacity for glucose transport results in increased glucose-6-phosphate (G-6-P) availability for utilization in glycolysis and the pentose phosphate pathway (PPP)^29^. Another study has shown that AKT phosphorylates and activates phosphofructokinase-2 (PFK2), which is the sole enzyme responsible for the production and degradation of fructose-2, 6-bisphosphate (F-2, 6-BP). F-2,6-BP is not directly involved in the glycolysis, but it allosterically activates PFK-1 more potently than its own product, fructose 1,6-bisphosphate (F-1,6-BP)^70^. By analogy with glycolysis, emerging evidence suggests that increased AKT may also affect oxidative metabolism. AKT indirectly promotes oxidative phosphorylation via increased formation of and access to glycolysis-derived substrates essential for TCA cycle activity and oxidative phosphorylation (e.g. pyruvate, ADP, NADH)^29^. CREB, phosphorylated by AKT at Ser133, is a key regulator of various intracellular processes including proliferation, differentiation, survival, neurogenesis, and neuronal plasticity^71^. CREB has been investigated as a metabolic sensor and regulator of glucose homeostasis in the HP^72^. CREB activation by synaptic activity induces the expression of the GLUT3 and promotes Siah2-mediated stabilization of hypoxia-inducible factor 1α that upregulates the expression of glycolysis genes^73^. In addition, previous studies have shown that NBP can exert actions through the AKT/CREB signaling pathway. L-3-n-butylphthalide (NBP) stimulates proliferation, migration, and differentiation of hippocampal neural stem cells in amyloid precursor protein/presenilin 1 (APP/PS1) mice by activating the AKT/CREB signaling pathway^74^. NBP promotes neurogenesis and neuroplasticity in cerebral ischemic rats via AKT–CREB signaling^75^. In the present study, we observed an increase in expression of p-AKT and p-CREB after NBP treatment in the HP, whereas expression of p-CREB was reduced in the PFC after NBP administration. Consequently, this may suggest that NBP alters energy metabolism in the HP and PFC via regulation of the AKT/CREB signaling pathway. A limitation of our study was the incomplete validation of the potential mechanism of NBP on depression, that is to say, the other signaling mechanisms involved in the effect of NBP on energy metabolism did not be included. On this basis, we may make investigation in further studies.

Together, NBP effectively attenuates CSDS-induced social deficits, anxiety-like behavior, and despair behavior. Targeted metabolomics profiling of the HP and PFC revealed that NBP alters metabolite levels of glycolysis and TCA cycle components. NBP also activated AKT and CREB phosphorylation to affect energy metabolism. Altogether, our results show that NBP can exert antidepressant effects by regulating energy metabolism via the AKT/CREB signaling pathway. Our findings provide the first evidence that NBP has antidepressant effects and yield important insights into the molecular energy metabolism changes underlying the therapeutic actions of NBP, thereby aiding research of novel antidepressant drugs.

## Supplementary information

supplementary materials
