# Supplementary material for: Dl-3-n-butylphthalide attenuates mouse behavioral deficits to chronic social defeat stress by regulating energy metabolism via AKT/CREB signaling pathway
Source: Transl Psychiatry. 2020 Feb 3;10:49. doi: 10.1038/s41398-020-0731-z (PMC7026059; doi:10.1038/s41398-020-0731-z)
Supplement: Supplementary file 1 — supplementary materials [file 41398_2020_731_MOESM1_ESM.docx]

**Supplementary Information**


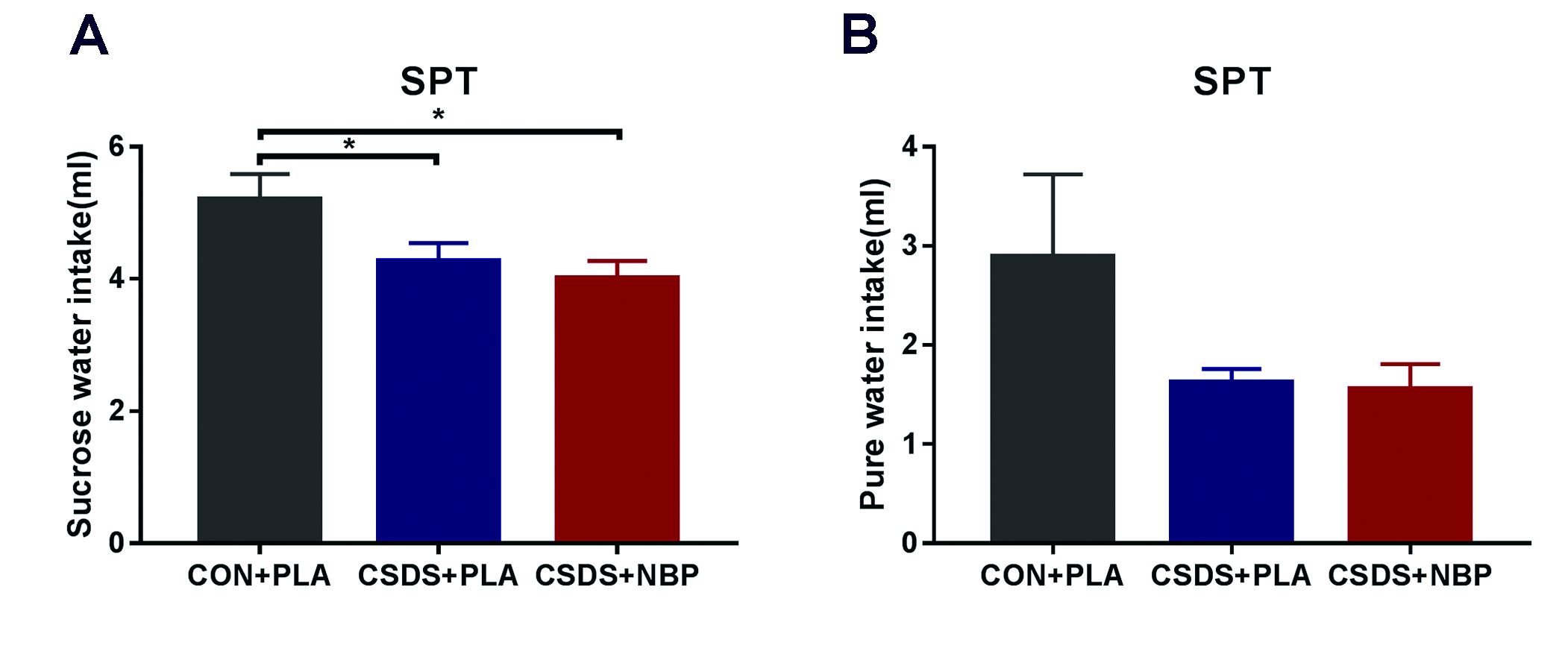


**Figure S1.** The sucrose water intake and pure water intake in sucrose preference test after chronic social defeat stress procedure and NBP treatment.

A) The sucrose water intake in sucrose preference test (SPT). B) The pure water intake in SPT. *p < 0·05. The data were represented as Mean ± SEM.


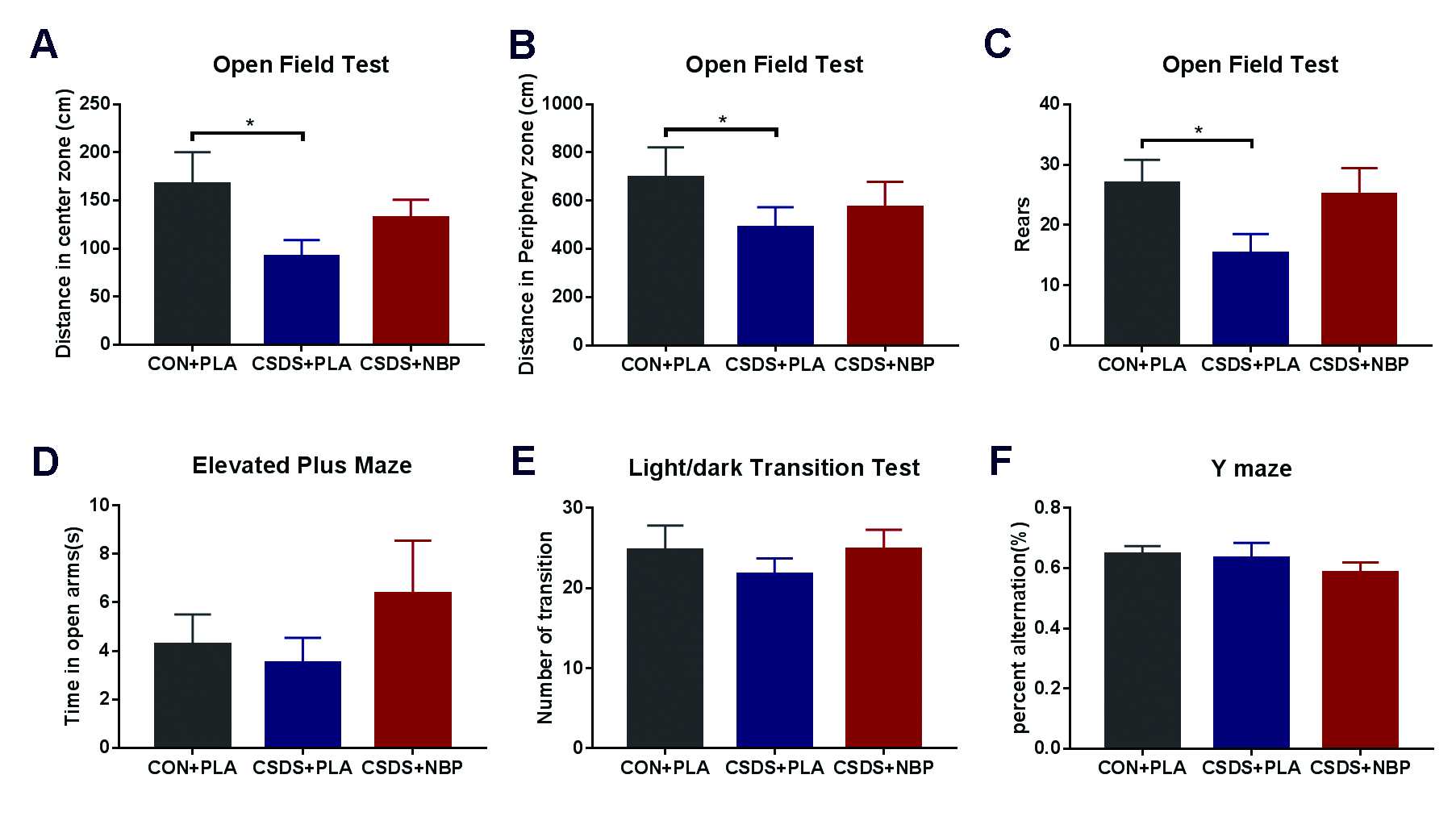


**Figure S2.** The effects of drug treatment and chronic social defeat stress procedure on the anxiety-like behavior and spatial working memory.

A) The distance in center zone in open field test (OFT). B) The distance in periphery zone in OFT. C) Rears in OFT. D) Time in open arms in elevated plus maze test. E) Number of transition in light/dark transition test. F) The percentage of alternations in the Y-maze. *p < 0·05. The data were represented as Mean ± SEM.

**
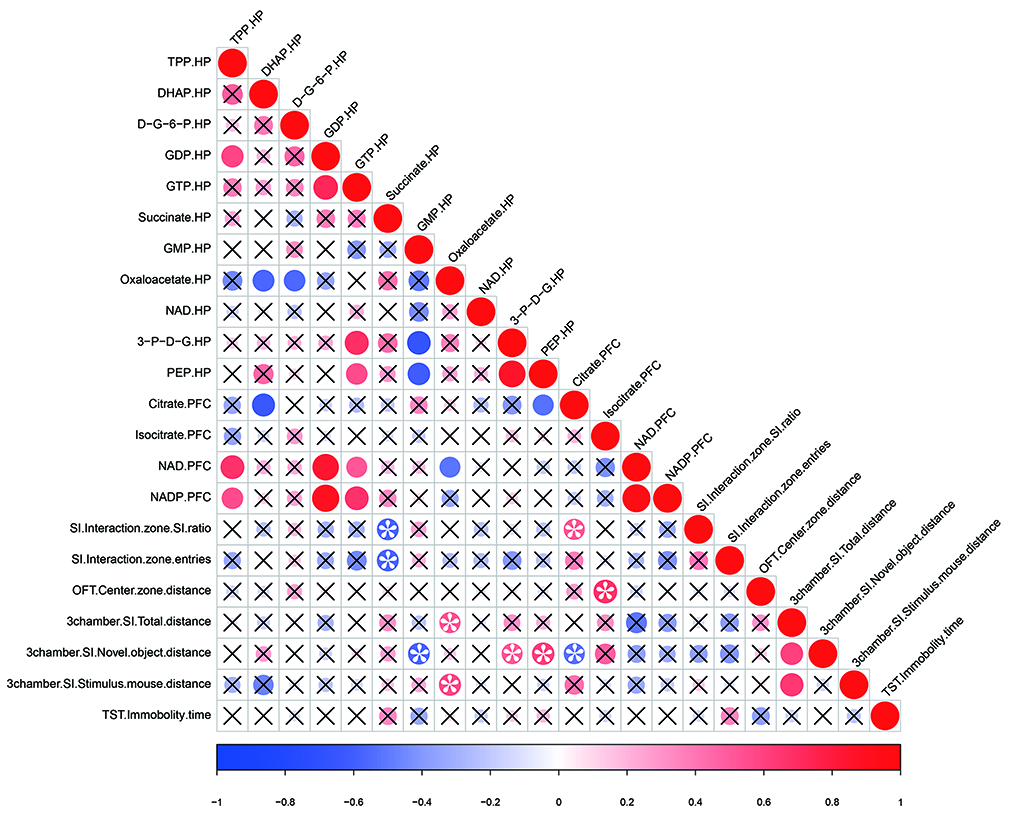
**

**Figure S3.** Target metabolites levels in the hippocampus and prefrontal cortex correlate with depressive behavior. The color and size of circles in the matrix code reflect the level of correlation: darker color and bigger circles reflect greater correlation. Red represents positive correlation and blue represents negative correlation. *p < 0.05.

**
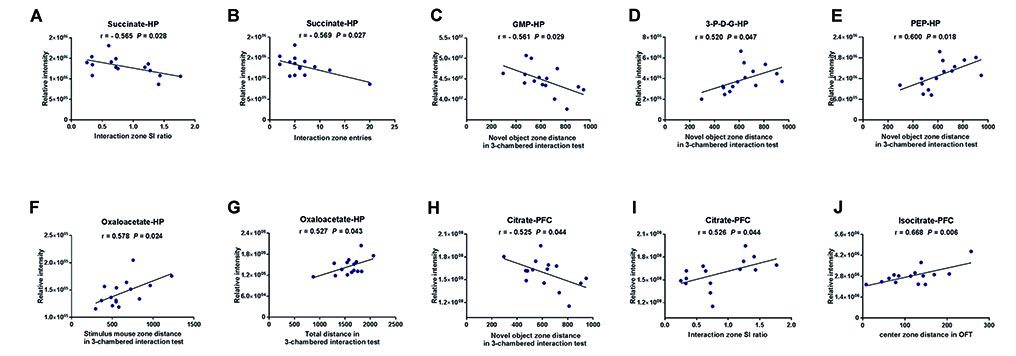
**

**Figure S4.** Correlation between altered metabolite levels in the hippocampus and prefrontal cortex and altered behavior in CSDS-treated mice. A-G) In the hippocampus (HP), succinate correlates with interaction zone social interaction (SI) ratio (A) and interaction zone entries (B). GMP (C), 3-P-D-G (D), and PEP (E) correlate with novel object zone distance in the 3-chambered test. Oxaloacetate correlates with stimulus mouse zone distance (F) and total distance (G) in the 3-chambered interaction test. H-J) In the prefrontal cortex (PFC), citrate correlates with novel object zone distance in the 3-chambered test (H) and interaction zone SI ratio (I). Isocitrate correlates with center zone distance in the open field test (J).


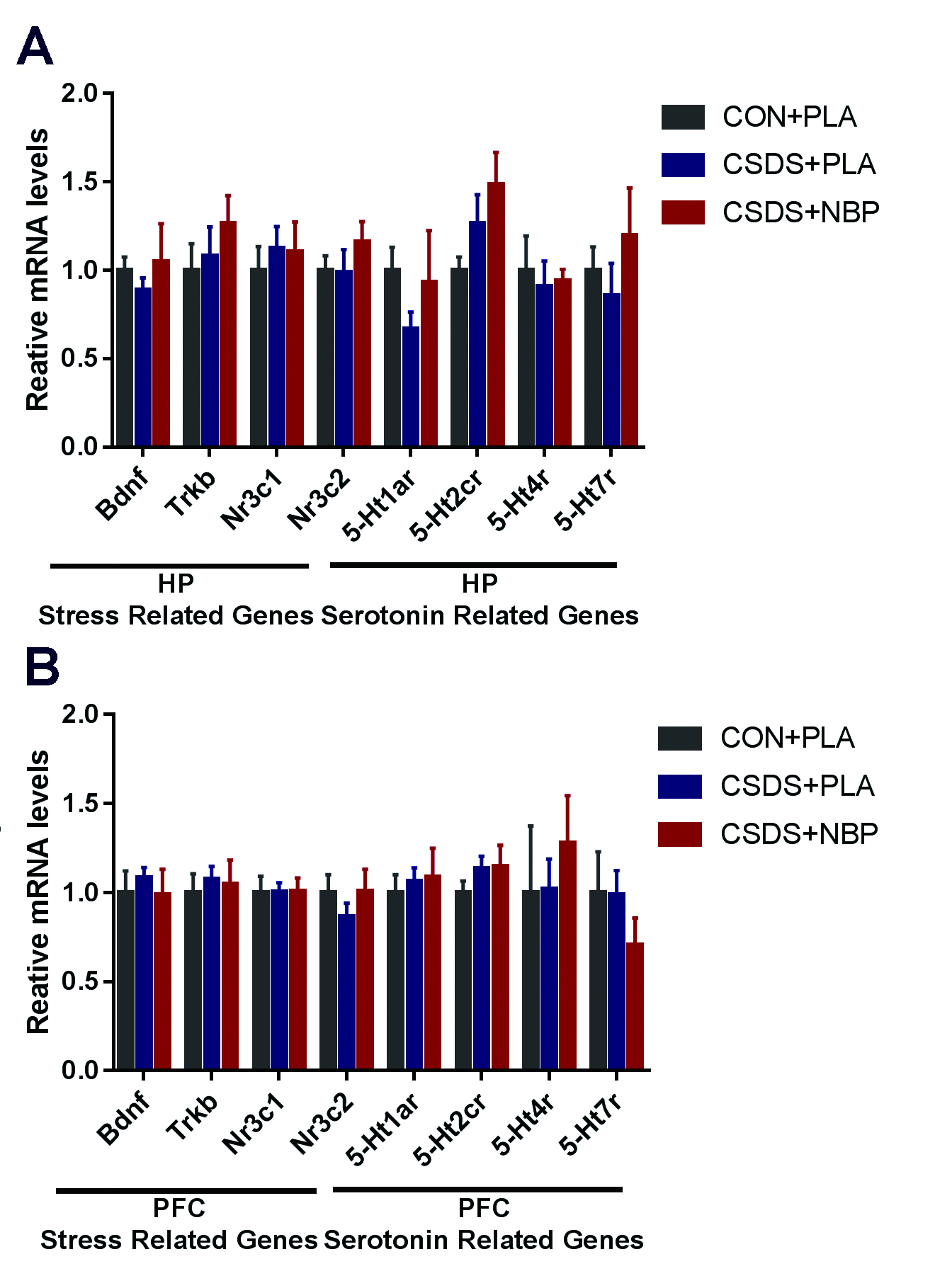


**Figure S5.** The effect of NBP treatment on mRNA expression levels of stress related genes and serotonin related genes in hippocampus and prefrontal cortex. The data were represented as Mean ± SEM.

**.**
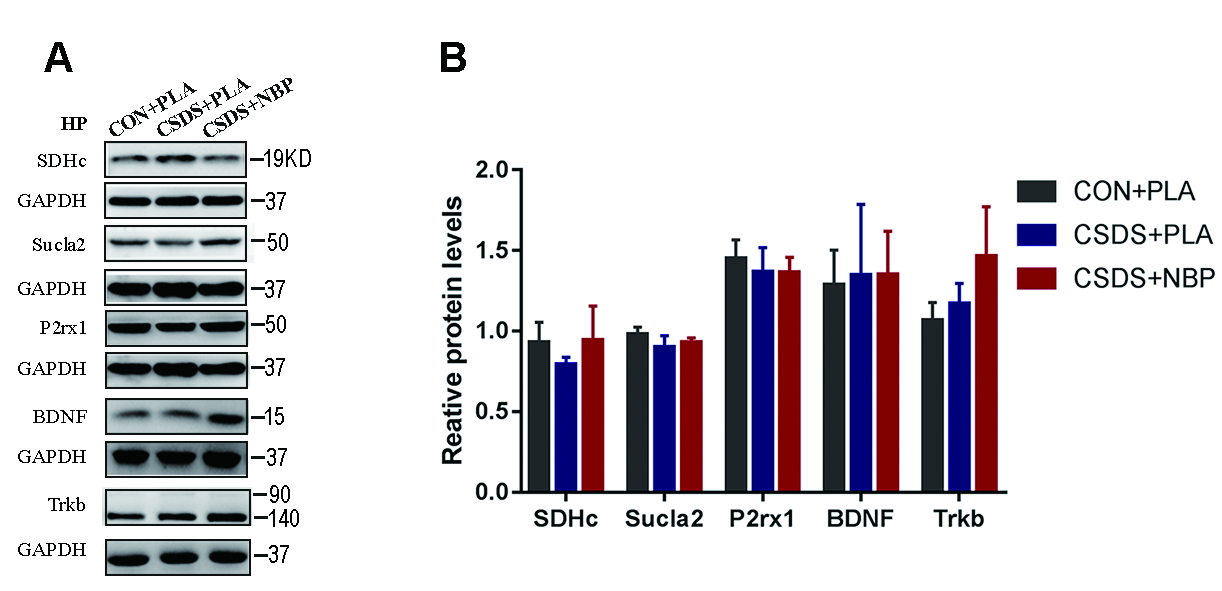


**Figure S6.** The effect of NBP treatment on protein expression levels of SDHc, Sucla2, P2rx1, BDNF and Trkb in hippocampus was detected by Western blotting. The data were represented as Mean ± SEM.


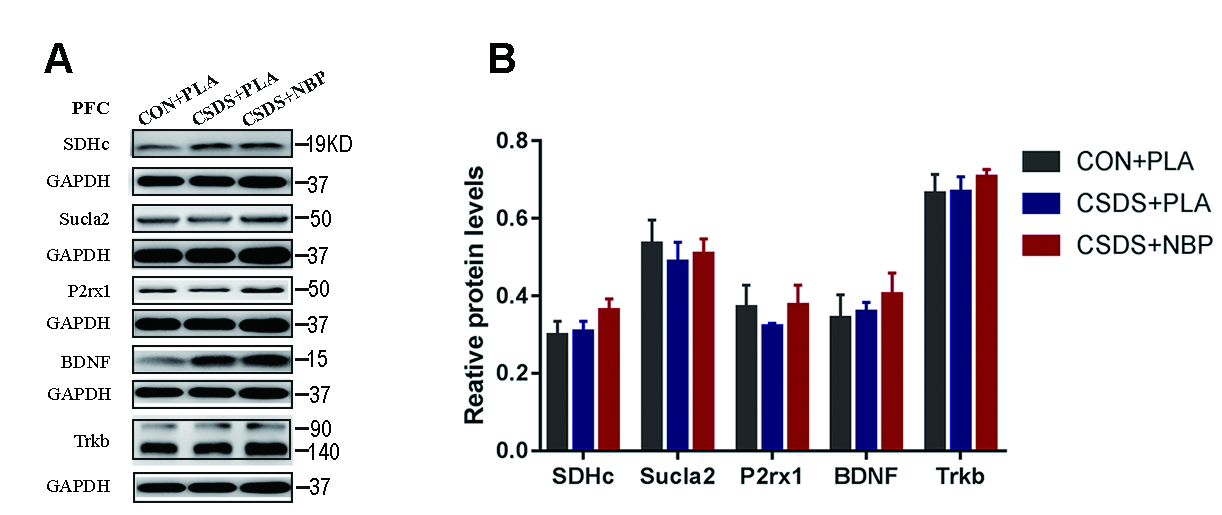


**Figure S7.** The effect of NBP treatment on protein expression levels of SDHc, Sucla2, P2rx1, BDNF and Trkb in prefrontal cortex was detected by Western blotting. The data were represented as Mean ± SEM.


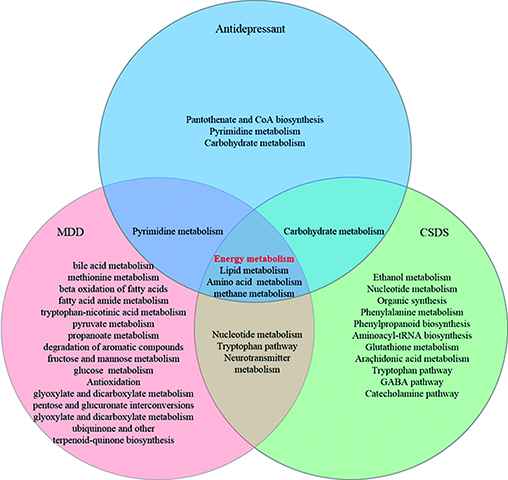


**Figure S8.** Differences and similarities of the disturbed metabolic pathways in MDD patients, CSDS model and after treatment. MDD, major depressive disorder; CSDS, chronic social defeat stress.

**Table S1.** Primer sequences for RT-qPCR

| Genes | Forward Primer (From 5′ to 3′) | Reverse Primer (From 5′ to 3′) |
| --- | --- | --- |
| *Bdnf* | TTACCTGGATGCCGCAAACAT | TGACCCACTCGCTAATACTGTC |
| *Trkb* | CTGGGGCTTATGCCTGCTG | AGGCTCAGTACACCAAATCCTA |
| *Akt* | ATGAACGACGTAGCCATTGTG | TTGTAGCCAATAAAGGTGCCAT |
| *Creb* | TCAGCCGGGTACTACCATTC | TTCAGCAGGCTGTGTAGGAA |
| *Nr3c1* | AGCTCCCCCTGGTAGAGAC | GGTGAAGACGCAGAAACCTTG |
| *Nr3c2* | GAAAGGCGCTGGAGTCAAGT | TGTTCGGAGTAGCACCGGAA |
| *5-Ht1ar* | GACAGGCGGCAACGATACT | CCAAGGAGCCGATGAGATAGTT |
| *5-Ht2cr* | GGTCCTTCGTGGCATTCTTCATC | CGCAGTTCCTCCTCGGTGTG |
| *5-Ht4r* | AGTTCCAACGAGGGTTTCAGG | CAGCAGGTTGCCCAAGATG |
| *5-Ht7r* | TGCGGGGAGCAGATCAACTA | GACAAAGCACACCGAGATCAC |
| *P2rx1* | CTGGTGGAGGAGGTGAAT | ATGGAACTGGTAGATGGGT |
| *P2rx2* | TGGAACTGTGACCTGGACT | GTGGTGCCGTTTATCTTG |
| *P2rx3* | ACAAGATGGAGAATGGCAGC | GCAGGATGATGTCACAGAGAAC |
| *P2rx4* | GACCAACACTTCTCAGCTTGG | GTGACGATCATGTTGGTCATG |
| *P2rx5* | GCCTATACCAACACCACGATG | CTTCACGCTCAGCACAGATG |
| *P2rx7* | GACAAACAAAGTCACCCGGAT | CGCTCACCAAAGCAAAGCTAAT |
| *Cs* | GGACAATTTTCCAACCAATCTGC | TCGGTTCATTCCCTCTGCATA |
| *Mdh* | CATTGGGCAACCCCTTTCAC | ACCTTTGAGGCAATCTGGCA |
| *Idh* | AAGGTTATGGCTCCCTTGGC | TAGTGACGTGTGACAGTGCC |
| *Sdha* | GGAGCAAATTCTCTCTTGGA | CAAATCTCAACTTGTCAAGATTC |
| *Sdhb* | AGCCTTATCTGAAGAAGAAGG | TACTTGTCTCCGTTCCACCAG |
| *Sdhc* | GAAGAACACGAGTTCAAACCG | AAAGTTCCCAGGAAGCAGCA |
| *Sucla1-GDP* | CCTCCTGCAACAGAATGGGA | GTGGTTCCTCCCACGAGTTT |
| *Sucla2-GDP* | CCCCGAAGATGGCTGAACC | ACCTCCTTTCAAACCGCTATTG |
| *Sucla2-ADP* | ACCCTTTCGCTGCATGAATAC | CCTGTGCCTTTATCACAACATCC |
| *β-actin* | AGAGGGAAATCGTGCGTGAC | CAATAGTGATGACCTGGCCGT |
| *Gapdh* | AGGTCGGTGTGAACGGATTTG | TGTAGACCATGTAGTTGAGGTCA |

**Table S2.** List of differential metabolites among three groups in mice HP and PFC

| **Brain region** | **Metabolites** | **Analyte Mass Range** | **RT/min** | **CSDS VS.CON** | | **NBP VS.CSDS** | | **NBP VS.CON** | |
| --- | --- | --- | --- | --- | --- | --- | --- | --- | --- |
|  |  |  |  | ***p-*Value** | **Fold change** | ***p-*Value** | **Fold change** | ***p-*Value** | **Fold change** |
| HP | Thiamine pyrophosphate (TPP) | 423·0 / 302·0 | 12·458 | 0·036 ^a^ | 1·13 |  |  | 0.031 | 1.14 |
|  | Dihydroxyacetone phosphate | 169·1 / 97·0 | 7·576 | 0·040 | 1·25 |  |  |  |  |
|  | D-Glucose 6-phosphate | 259·0 / 97·0 | 9·284 | 0·012 | 0·85 |  |  |  |  |
|  | Guanosine 5'-diphosphate (GDP) | 442·0 / 79·0 | 10·072 | 0·032 | 1·30 |  |  |  |  |
|  | Guanosine 5'-triphosphate (GTP) | 522·0 / 159·1 | 11·144 | 0·020 | 1·33 | 0·003 | 0·65 |  |  |
|  | Succinate | 117·1 / 73·0 | 5·964 | 0·037 | 1·21 | 0·032 | 0·82 |  |  |
|  | NAD | 662·1 / 540·0 | 8·113 |  |  | 0·013 | 0·77 |  |  |
|  | Phosphoenolpyruvate | 167·1 / 79·0 | 8·518 |  |  | 0·016 | 0·63 |  |  |
|  | GMP | 362·1 / 79·1 | 8·906 |  |  | 0·013 | 1·12 | 0·019 | 1·11 |
|  | Oxaloacetate | 131·1 / 87·1 | 5·798 |  |  | 0·007 | 0·84 | 0·011 | 0·75 |
|  | 3-Phospho-D-glycerate | 185·0 / 97·0 | 8·737 |  |  | 0·015 | 0·60 | 0·030 | 0·63 |
| PFC | Citrate | 191·1 / 87·1 | 8·782 | 0·032 | 0·85 |  |  |  |  |
|  | Isocitrate | 191·0 / 73·0 | 8·541 | 0·017 | 0·76 |  |  | 0·013 | 0·75 |
|  | NAD | 662·1 / 540·0 | 7·250 | 0·002 | 2·06 |  |  | 0·005 | 1·94 |
|  | NADP | 742·1 / 620·0 | 10·255 | 0·009 | 1·61 |  |  |  |  |

CON, CON+PLA group; CSDS, CSDS+PLA group; NBP, CSDS+NBP group; HP, hippocampus; PFC, prefrontal cortex.

^a^ one-way analysis of variance (ANOVA).

p<0.05.
